# Evaluation of the association between tongue posture and the transverse dental compensation in Class III skeletal patients – a retrospective, record-based study

**DOI:** 10.4317/jced.62106

**Published:** 2025-08-01

**Authors:** Mayithiri Balaji, Ashith M-V, Siddharth Shetty, Amoli Singh, Harshit Atul Kumar, Rainee Solanki

**Affiliations:** 1 Post Graduate Student,Department of Orthodontic and Dentofacial Orthopedics, Manipal College of Dental Sciences Mangalore, Affiliated to Manipal Academy of Higher Education, Manipal, Karnataka, India; 2 Professor and Head, Department of Orthodontic and Dentofacial Orthopedics, Manipal College of Dental Sciences Mangalore, Affiliated to Manipal Academy of Higher Education, Manipal, Karnataka, India; 3 Orthodontic Practitioner, First Floor, Medicare Centre, Bunts Hostel, Karangalpady, Mangalore, India; 4 Senior Lecturer, Department of Orthodontic and Dentofacial Orthopedics,Manipal College of Dental Sciences Mangalore,Affiliated to Manipal Academy of Higher Education, Manipal, Karnataka, India

## Abstract

**Background:**

To assess the association between tongue posture and transverse dental compensation patterns in teeth of Class III skeletal patients and to compare the above associations between Class III skeletal patients and Class I skeletal patients.

**Material and Methods:**

This retrospective study was conducted in the Department of Orthodontics and Dentofacial Orthopedics, Manipal College of Dental Sciences, Mangalore. The records of 126 subjects including skeletal class I subjects as the control group (n = 63) and skeletal class III patients as the experimental group (n = 63) were obtained. The tongue-to-palate distance of all the subjects was measured using the method described by Graber (Graber *et al*., 1997) on lateral cephalograms. CBCT images were standardized and acquired by keeping the patient’s head in the natural position. Buccolingual inclinations were measured on CBCT coronal sections as the angle between the reference plane (line perpendicular to the line joining the buccal and lingual crest of the alveolar bone) and the long axis of each tooth. SPSS software, version 25.0 was used to perform the statistical analyses. To analyze the association between tongue posture and buccolingual inclination of teeth, the Pearson correlation coefficient was calculated, with a power of 80%.

**Results:**

Statistically significant differences were found for skeletal Class III malocclusion. The degree of buccal tipping at 17, 15, 14, 27, 25, 24 and the tongue-to-plate distance at D3 and D4 were significantly correlated, with a *p value* <0.001. The degree of buccal tipping at 16, 26 and the tongue-to-plate distance at D1, D2, D3, D4 and D5 showed a moderate positive correlation and was significant. In general, a positive correlation was observed between the degree of buccal tipping of maxillary posterior teeth and the tongue posture at D1–D5. A moderate positive correlation was observed between the degree of lingual tipping of molars and premolars and the tongue-to-palate distance at D1, D2, D3, D4 and D5.

**Conclusions:**

Based on the observations of skeletal Class III patients, the following conclusions can be drawn:

a) The tongue assumes a lower and more anterior position than does the tongue in Class I subjects.

b) The tongue-to-palate distance increases at positions D2–D5, revealing a strong association between tongue forces and buccal flaring of maxillary posterior teeth.

c) Lingual tipping of mandibular posterior teeth showed a constant moderate association with tongue position at D1–D5 and was significant, but not as significant as that observed for maxillary posterior teeth.

** Key words:**Tongue posture, buccolingual tooth inclination, skeletal class III, dentoalveolar compensation, soft tissues.

## Introduction

Skeletal Class III malocclusion is characterized by mandibular prognathism, maxillary deficiency, or a combination of the two features. Genetic and/or environmental factors impact the growth of the maxilla and mandible [1,2].

Similar studies have revealed that the incidence of class III malocclusion and underlying skeletal types vary among racial and ethnic groups, as well as within the same ethnic population [3].

With the presence of a skeletal base discrepancy, transverse discrepancy is more often found in Class III skeletal patients; therefore, the buccal inclinations of posterior maxillary teeth are considered a compensatory mechanism for dentoalveolar movement [4]. This complex process is multifactorial and includes anteroposterior and vertical facial skeletal patterns [5]. The causes of this complication are multifactorial and commonly involve an underdeveloped maxilla with an overdeveloped mandible and a low tongue posture.

It has been established that form and function are strongly associated and important factors that affect dental compensation in Class III skeletal patients, via the use of soft tissues. Soft tissues play a role in establishing the form, position, growth and maintenance of skeletal tissues and organs. Therefore, the growth of skeletal tissues and organs is a result of secondary compensatory and necessary responses (Moss, 1981) [6].

The tongue is a powerful muscular organ that affects the position of teeth and surrounding structures. Many orthodontists have endorsed the concept of labio-lingual muscle force balance. Kydd *et al*. [7] reported that the tongue, during rest and function, exerts greater muscular force than the lips and cheeks. The importance of muscles in preserving the arch form and position of teeth was noted. Hence, the tongue plays a significant role in this process, and understanding the underlying mechanism is key when planning appropriate treatments.

Tongue posture is extremely useful for assessing the effect of tongue posture on the development of Class III skeletal malocclusion as well as its relation to the buccolingual inclinations of teeth. Hence, a relationship between tongue posture (tongue height measured as the tongue-to-palate distance), and the buccolingual inclinations of teeth must be established [8]. Therefore, transverse dental compensation affects all posterior teeth, and the pattern of compensation differs [9]. Therefore, it is essential to view the compensation occurring in premolars as well.

Earlier studies evaluated tongue posture only with dentoalveolar compensation from a two-dimensional aspect, which view the tongue-to-palate distance without considering this association to tooth inclinations. Cone-beam computed tomography (CBCT) has allowed the assessment of the buccolingual inclination of crowns and roots as well as mesiodistal angulation with respect to the alveolar bone crest. In this study, CBCT sections determined the degree of buccal and lingual tipping of maxillary and mandibular posterior teeth respectively. Hence, clear correlation between the tongue-to-palate distance and tooth inclinations were attained.

As a result, tooth inclination is influenced by dental adaptation or as a compensatory factor for skeletal discrepancies. The above evaluation could contribute to a more detailed treatment plan. Therefore, if the underlying cause is not addressed, relapse may occur owing to the manifestation of the actual growth pattern and aberrant muscular forces.

Hence, this study was planned and designed with the objective of assessing and comparing the effect of tongue posture on buccolingual inclinations of teeth in individuals with a prognathic and an orthognathic mandible so to help rectify the nature of the tongue to attain proper inclinations of teeth as well as establishing a proper and stable occlusion.

The aim of the present study was to evaluate the association between tongue posture and transverse dental compensation patterns of teeth in Class III skeletal patients and to compare the above associations between Class III skeletal patients and Class I skeletal patients.

Aims:

1) To evaluate the association between tongue posture (obtained from lateral cephalogram) and transverse dental compensation patterns in teeth (obtained from CBCT sections) in Class III skeletal patients

2) To compare the above associations between skeletal Class III patients and skeletal Class I patients

## Material and Methods

This was a retrospective, record-based study, conducted in the Department of Orthodontics and Dentofacial Orthopedics, Manipal College of Dental Sciences, Mangalore. The present study was approved by the “Institutional Ethics Committee” of the Manipal College of Dental Sciences, Mangalore (Ref No: 20087). Accordingly, 126 subjects were divided into groups: skeletal class III (group I – experimental group) and skeletal class I (group II - control group). The criteria for the experimental group were as follows:

Inclusion criteria:

1) Subjects older than 18 years and without any previous orthodontic treatment

2) Subjects with ANB angles less than 0° but not more than -3°

3) Patients with fully erupted permanent premolars and molars.

Exclusion criteria:

1) Patients with ectopic tooth eruption, missing/extracted teeth, crowding of teeth or any associated pathology or systemic disease

2) Patients with cleft lip/palate or any temporomandibular joint disease

3) Subjects with missing adult teeth (except 3rd molars) or with class III malocclusion

4) Subjects with severe crowding that require extraction.

The control group was selected according to the following criteria:

1) ANB between 1° and 4° and a Me deviation less than 2 mm

2) Anterior crowding less than 4 mm

3) No buccal or lingual crossbite of posterior teeth

4) The other criteria were the same as above.

**Figure 1:**
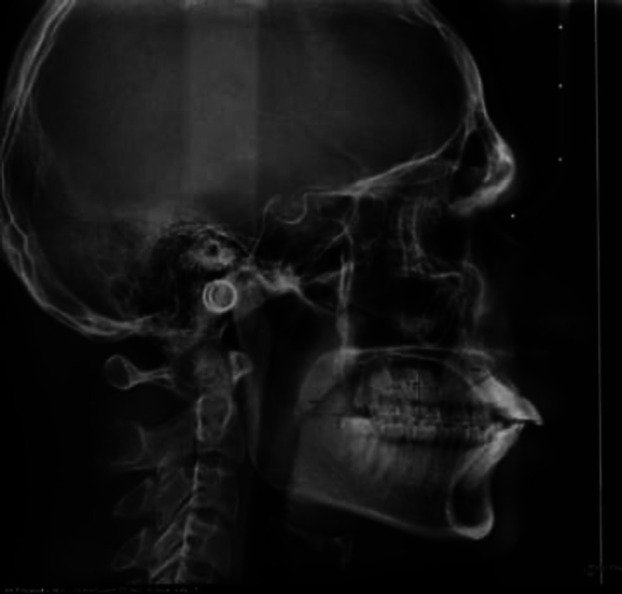
Assessment of tongue position via lateral cephalogram.

In assessing tongue posture, using the method described by Graber (Graber *et al*., 1997), as shown in the below mentioned Figure (Fig. 1), tongue-to-palate distances were measured on lateral cephalograms. A template with an inscribed millimeter scale was superimposed on the lateral cephalogram with its horizontal line passing through the incisal edge of the lower central incisor and the cervical distal third of the last erupted molar and extending to the most inferior point of the uvula.

Contours of the dorsum of the tongue and the bony palate were marked, and distances from point zero were measured at four different angles. The template was then placed at the midpoint of the horizontal line.

A further four lines were drawn at 30° to each other, resulting in a total of seven lines. From here, a perpendicular line was drawn to the roof of the mouth.

• Measurement made along line 1- Distance between the soft palate and the root of the tongue (posterior border of the oral cavity)

• Measurements were made along lines 2-6, the relationship between the dorsum of the tongue and the roof of the mouth.

• Along line 7- Position of the tip of the tongue relative to the lower incisor.

**Figure 2:**
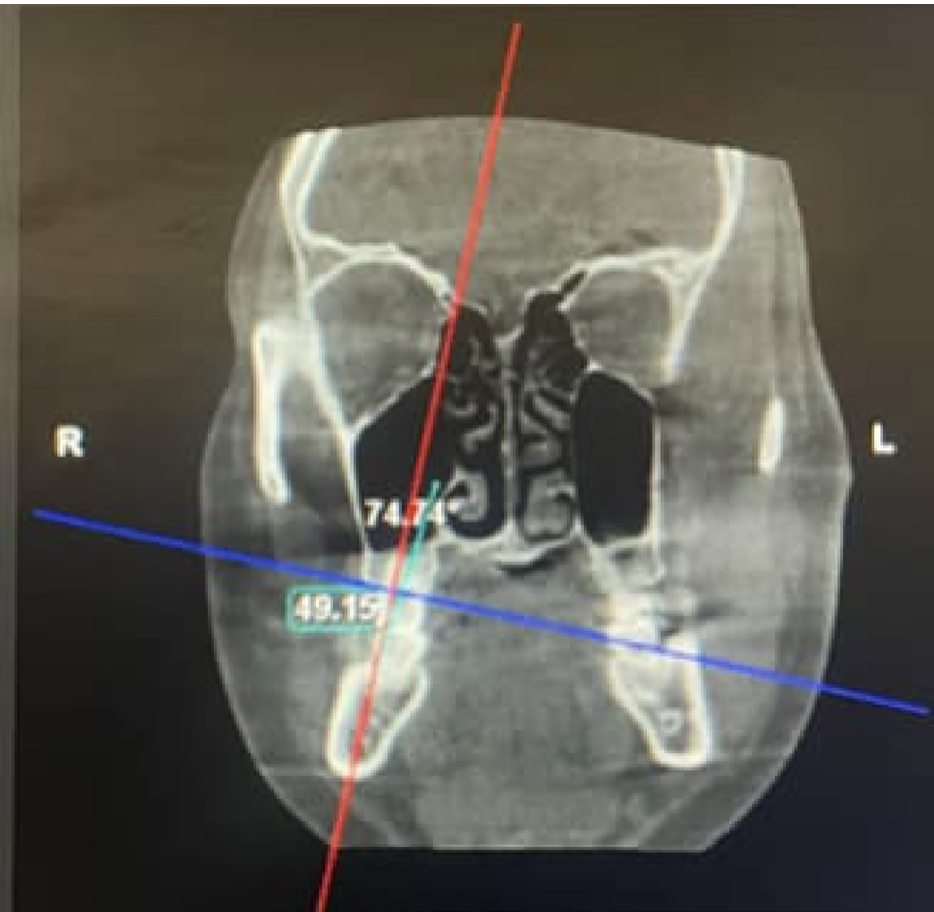
Coronal view of 46.

All the CBCT images were standardized (90 Kilovolt; at 5.6 milliamperes; voxel size 600 μm). All scans were performed by keeping the patient’s head in the natural head position. CBCT images were taken for patients as a diagnostic aid for their orthodontic treatment, strictly conforming to the Planmeca Ultra Low DoseTM (Planmeca ProMax® 3D Mid CBCT machine, manufactured by PLANMECA OY corporation, in Finland) imaging protocol, which facilitates 3D imaging at much lower levels of radiation than does standard 2D panoramic imaging. CBCT images in (Digital Images and Communications in Medicine (DICOM) format were opened in Planmeca Romexis Viewer 5.1.0 software for obtaining the measurements. The reconstructed 3D images were oriented as follows:

• In axial view, the image was adjusted so that the sagittal reference line (blue line) in the viewer was a line passing through the tooth showing both buccal and lingual alveolar crests in coronal section.

• In the coronal view, the reference plane (red line) is a line perpendicular to the line joining the buccal and lingual crest of the alveolar bone (blue line).

• In the coronal view, the second line (blue line) passes through the long axis of the tooth. The buccolingual inclinations were then measured as the angle between the reference plane and the long axis of each tooth, as shown in Fig. 2.

- Statistical analysis

The required sample size was 63 per group, for a total of 126 participants. SPSS software, version 25.0, was used to perform the analyses. Therefore, to further analyze the associations between tongue posture and buccolingual inclination of the teeth, the Pearson correlation coefficient was calculated.

**Figure 3:**
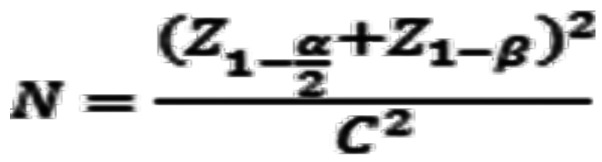
Formula.

With an alpha error of 5% and a power of 80%, the Z values of the given alpha and beta values are 1.95996398454005 and 0.841621233572915, respectively. With the correlation coefficient and using the above formula, the required sample size is 63, (Fig. 3).

N = No. of subjects

r = correlation coefficient; C = 0.5*ln[(1+|r|)/(1-|r|)]; r = -0.34; C = -0.35409; alpha error = 5%; Z(1-α/2) = 1.959964; power (1-β) = 80.00%; Z(1-β) = 0.841621

## Results

In skeletal Class 1 patients, when comparing the degree of buccal tipping of maxillary posterior teeth and tongue-to-palate distance, a negative correlation was mostly observed, which indicated that as one parameter increased, the other decreased. The positive correlation between the parameters found in skeletal Class I patients is shown in  Table 1.

As shown in  Table 1, there was a moderate positive correlation between the degree of buccal tipping at 16 and the Tongue-to-Palate distance at D7, which was significant, with a *p value* of 0.049. The correlation between the degree of buccal tipping at 27 and the Tongue-to-Palate distance at D7 was moderate and positive, which was significant, with a *p value* of 0.033. The correlation between the degree of lingual tipping of 47 and the tongue-to-palate distance at D7 was moderate and positive, which was significant, with a *p value* of 0.049.

In skeletal Class III patients (experimental group), when comparing the degree of buccal tipping of maxillary posterior teeth and tongue-to-palate distance, a positive correlation was mostly observed, which indicated that as one parameter increased, the other also increased. A significant positive correlation was observed for the tongue-to-palate distance from D1 to D5. No correlation was observed at positions D6 or D7.

 Table 2 compare the degree of buccal tipping of the maxillary posterior teeth in the 1st quadrant with the tongue-to-palate distances at D1, D2, D3, D4, and D5.

As shown in the above Table, the degree of buccal tipping of 17 and tongue-to-palate distances at D3 and D4 was strongly positively correlated, which was significant, with a *p value* <0.001. The correlations between the degree of buccal tipping of 17 and the tongue-to-palate distance at D1, D2 and were moderately positive and significant, with *p value*s of 0.003, 0.004 and 0.005, respectively.

In the above Table, the correlations between the degree of buccal tipping in 16 and the tongue-to-palate distance at D1, D2, D3, D4 and D5 were moderately positive and significant, with *p value*s of 0.008, 0.02, 0.002, 0.003 and 0.044, respectively.

As shown in the above Table, the correlation between the degree of buccal tipping at position 15 and the tongue-to-palate distance at D3 and D4 was good and was significant, with a *p value* <0.001. Correlations between the degree of buccal tipping of tooth 15 and the tongue-to-palate distance at D1, D2 and D5 were moderately positive and significant, with *p value*s of 0.002, 0.002 and 0.005, respectively.

As shown in the above Table, the correlations between the degree of buccal tipping of tooth 14 and the tongue-to-palate distance at D1, D2, D3 and D4 were positive and significant, with *p value*s of 0.001, 0.001, <0.001, and <0.001, respectively. The correlation between the degree of buccal tipping of 14 and the tongue-to-palate distance on D5 was moderate and positive and was significant, with a *p value* of 0.004.

 Table 3 compare the degree of buccal tipping of the maxillary posterior teeth in the 2nd quadrant with the tongue-to-palate distances at D1, D2, D3, D4, and D5. A significant positive correlation was observed for the tongue-to-palate distance from D1 to D5. No correlation was observed at positions D6 or D7.

As shown in the above Table, the correlation between the degree of buccal tipping at 27 and the tongue-to-palate distance at D3 and D4 was good and was significant, with a *p value* <0.001. The correlations between the degree of buccal tipping of 27 and the tongue-to-palate distance at D1, D2, and D5 were moderately positive and significant, with *p value*s of 0.004, 0.004, and 0.006, respectively.

As shown in the above Table, the correlations between the degree of buccal tipping of tooth 26 and the tongue-to-palate distance at D1, D2, D3, D4 and D5 were moderately positive and were significant, with *p value*s of 0.015, 0.034, 0.004, 0.004, and 0.044, respectively.

As shown in the above Table, the correlations between the degree of buccal tipping of tooth 25 and the tongue-to-palate distance at D1, D2, D3, and D4 were positive and significant, with *p value*s of 0.001, 0.001, <0.001, and <0.001 respectively. The correlation between the degree of buccal tipping of tooth 25 and the tongue-to-palate distance at D5 was moderate and positive and was significant, with a *p value* of 0.002.

As shown in the above Table, the correlations between the degree of buccal tipping at position 24 and the tongue-to-palate distance at D1, D2, D3, D4 and D5 were positive and significant, with *p value*s <0.001, <0.001, <0.001, <0.001, and 0.001, respectively.

As shown in  Table 4 and  Table 5, there was a positive correlation between the degree of lingual tipping of mandibular posterior teeth in the 3rd and 4th quadrants and the tongue-to-palate distance at D1, D2, D3, D4, and D5. No correlation was observed at positions D6 or D7.

In the above Table, the correlations between the degree of lingual tipping of tooth 37 and the tongue-to-palate distance at D1, D2, D3, D4 and D5 were moderately positive and significant, with *p value*s of 0.007, 0.013, 0.004, 0.006, and 0.041, respectively.

In the above Table, the correlations between the degree of lingual tipping of tooth 36 and the tongue-to-palate distance at D1, D2, D3, D4 and D5 were moderately positive and significant, with *p value*s of 0.009, 0.015, 0.006, 0.011, and 0.04, respectively.

In the above Table, the correlations between the degree of lingual tipping in 35 and the tongue-to-palate distance at D1, D2, D3, D4 and D5 were moderately positive and significant, with *p value*s of 0.01, 0.015, 0.005, 0.008, and 0.28, respectively.

In the above Table, 34 and tongue-to-plate distances at D1, D2, D3, D4 and D5 were moderately positively correlated and significantly correlated, with *p value*s of 0.008, 0.015, 0.007, 0.01, and 0.032, respectively.

In the above Table, the degrees of lingual tipping of 47 and tongue-to-palate distances at D1, D2, D3, and D4 were moderately positively correlated and were significantly correlated, with *p value*s of 0.01, 0.01, 0.008, and 0.006, respectively.

In the above Table, the correlations between the degree of lingual tipping in 46 and the tongue-to-palate distance at D1, D2, D3, D4 and D5 were moderately positive and were significant, with *p value*s of 0.005, 0.013, 0.003, 0.013 and 0.048, respectively.

In the above Table, the degrees of lingual tipping at 45 and tongue-to-plate distance at D1, D2, D3, and D4 were moderately positively correlated and were significantly correlated, with *p value*s of 0.01, 0.02, 0.007, and 0.013, respectively.

In the above Table, the correlations between the degree of lingual tipping of tooth 44 and the tongue-to-plate distance at D1, D2, D3, D4 and D5 were moderately positive and were significant, with *p value*s of 0.012, 0.023, 0.007, 0.013 and 0.043, respectively.

## Discussion

According to a review of the orthodontic literature, the soft tissue forces and balance of the oral musculature have not been well understood. Soft tissue pressure plays an essential role in maxillary-mandibular development, and tongue forces play a significant role in tooth eruption, development of the dental arch, and stability. In addition, the resting tongue position is linked to sagittal jaw relationships [10]. Additionally, it is important to assess the impact of these forces on dentition, as buccolingual tooth inclination is part of the Andrews Six Keys to Normal Occlusion and is extremely important for establishing better treatment outcomes [11].

Hence, the purpose of the present study was to assess the relationship between forces exerted by the tongue on the buccolingual inclinations of maxillary and mandibular posterior teeth. The tongue is incredibly adapTable to its surroundings. Swinehart *et al*. [12] reported that the most important natural force that increases the mandibular arch dimension is the tongue. Some mandibular arches are too narrow for accommodating the tongue; therefore, the tongue exerts expansive forces. The resulting and coordinated forces of the tongue, cheek and lips, when allowed to reach proper equilibrium during the early stages of facial growth, usually maintain proper balance.

In the present study, the tongue-to-palate distances were evaluated according to the method described by Graber (Graber *et al*., 1997) [13], and most of the differences in terms of tongue-to-palate distance were observed at the posterior regions (measurement points 2–5) with regard to Class III skeletal patients. In addition, buccolingual inclinations of the posterior teeth were assessed using a line passing through the tooth, which shows a clear view of the buccal and lingual alveolar crests in the coronal CBCT section. In previous studies performed by Shewinvanakitkul *et al*. [14], the plane of reference used was the mandibular plane. Even though the mandibular plane is less likely to be influenced by orthodontic tooth movement, it is still variable.

In a previous study performed by Kim *et al*. [4], dentoalveolar compensation for the anterior segment was analyzed, with no regard given to the posterior segment. A study by Ahn *et al*. [15] demonstrated that sagittal skeletal discrepancy mainly affects the buccolingual inclination of maxillary teeth. Compared with Class I patients, Class III skeletal patients had more buccally inclined maxillary posterior teeth, and this difference was negatively correlated with the ANB angle, indicating that a sagittal skeletal discrepancy is an important cause of transverse discrepancy.

In the present study, regarding skeletal Class I subjects, the only significant positive correlations found between the parameters were 1) the degree of buccal tipping of maxillary posterior teeth 16, 27 and the tongue-to-palate distance at D7 and 2) the degree of lingual tipping of 47 and the tongue-to-palate distance at D7. Therefore, the tongue at rest exerts its innate forces, which mold the dentition accordingly.

In this study, regarding skeletal Class III patients, between the degree of buccal tipping (17,15,27,14,25 and 24) and the tongue-to-palate distance at D3 and D4 showed good positive correlation with a significant *p value* <0.001. The maxillary posterior teeth tend to tip buccally maximally at tongue-to-palate distances D3 and D4. This finding shows that as the tip of the tongue is anteriorly positioned, it assumes a lower position on the floor of the mouth in patients with a prognathic mandible. The distance between the tongue and the hard palate increases, which in turn leads to an increase in the size of the posterior portion of the tongue, whereas the anterior portion is less bulky and more anteriorly positioned.

Forces of the tongue push the maxillary posterior teeth outwards, and due to an unbalanced force of the cheek, mandibular posterior teeth tend to incline lingually.

The facial muscles as well as the muscles of mastication create a greater force, thereby resisting the effect of tongue forces on mandibular dentition. As the forces produced by soft tissues were not studied in this study, further studies could be performed to evaluate the forces individually produced by the cheek and tongue.

In the present study, skeletal Class III patients showed a moderate positive correlation between the degree of lingual tipping of molars and premolars and the tongue-to-palate distance at D1, D2, D3, D4 and D5. This may indicate that the buccinator mechanism, rather than the tongue mechanism, exerts more effective pressure on the buccolingual positioning of the mandibular molars.

In this study, it is important to note that the exclusion criterion comprised individuals in whom the association between the transverse relationship and tongue-to-palate distance could be compromised. The study also does not take gender variation into account. The tongue position was assessed via two-dimensional imaging. Additionally, measuring tongue position, particularly in the context of a closed oral cavity, poses inherent challenges due to the dynamic nature of the tongue. The considerable variability associated with capturing tongue-to-palate distances during the film acquisition process further poses as a drawback of this study.

Therefore, conclusions cannot be drawn from a single study, and additional research should be performed to demonstrate a cause-and-effect link.

## Conclusions

The following conclusions can be drawn from the current study:

Based on the observations in skeletal Class III patients, the following conclusions can be drawn:

a) The tongue assumes a lower and more anterior position than does the tongue in Class I subjects.

b) The tongue-to-palate distance increases at positions D2–D5, revealing a strong association between tongue forces and buccal flaring of maxillary posterior teeth.

c) Lingual tipping of mandibular posterior teeth showed a constant moderate association with tongue position at D1–D5 and was significant but not as significant as that observed for maxillary posterior teeth. This finding suggested that the concept of ideal occlusion, wherein the lingual inclination from premolars to molars progressively increased in the mandibular teeth, as described by Andrews *et al*., was maintained.

## Figures and Tables

**Table 1:** Correlations between the degree of buccal tipping at 16 and 27 and the degree of lingual tipping at 47 and tongue-to-palate distances at D7.

PARAMETERS BEING CORRELATED	N	Correlation®	P VALUE
Degree of buccal tipping of 16 & Tongue to Palate distance at D7	63	0.249	0.049
Degree of buccal tipping of 27 & Tongue to Palate distance at D7	63	0.27	0.033
Degree of lingual tipping of 47 & Tongue to Palate distance at D7	63	0.249	0.049

**Table 2:** Correlations between the degree of buccal tipping of tooth 17,16,15,14 and the tongue-to-palate distance at D1-D5.

Table 2 (A) Correlations between the degree of buccal tipping of tooth 17 and the tongue-to-palate distance at D1-D5			
PARAMETERS BEING CORRELATED	N	Correlation®	P VALUE
Degree of buccal tipping of 17 & Tongue to Palate distance at D1	63	0.368	0.003
Degree of buccal tipping of 17 & Tongue to Palate distance at D2	63	0.361	0.004
Degree of buccal tipping of 17 & Tongue to Palate distance at D3	63	0.451	<0.001
Degree of buccal tipping of 17 & Tongue to Palate distance at D4	63	0.466	<0.001
Degree of buccal tipping of 17 & Tongue to Palate distance at D5	63	0.349	0.005
Table 2 (B) Correlations between the degree of buccal tipping at position 16 and the tongue-to-palate distance at D1-D5			
PARAMETERS BEING CORRELATED	N	Correlation®	P VALUE
Degree of buccal tipping of 16 & Tongue to Palate distance at D1	63	0.333	0.008
Degree of buccal tipping of 16 & Tongue to Palate distance at D2	63	0.293	0.02
Degree of buccal tipping of 16 & Tongue to Palate distance at D3	63	0.38	0.002
Degree of buccal tipping of 16 & Tongue to Palate distance at D4	63	0.365	0.003
Degree of buccal tipping of 16 & Tongue to Palate distance at D5	63	0.255	0.044
Table 2 (C) Correlations between the degree of buccal tipping at position 15 and the tongue-to-palate distance at D1-D5			
PARAMETERS BEING CORRELATED	N	Correlation®	P VALUE
Degree of buccal tipping of 15 & Tongue to Palate distance at D1	63	0.388	0.002
Degree of buccal tipping of 15 & Tongue to Palate distance at D2	63	0.386	0.002
Degree of buccal tipping of 15 & Tongue to Palate distance at D3	63	0.479	<0.001
Degree of buccal tipping of 15 & Tongue to Palate distance at D4	63	0.484	<0.001
Degree of buccal tipping of 15 & Tongue to Palate distance at D5	63	0.351	0.005
Table 2 (D) Correlations between the degree of buccal tipping at position 14 and the tongue-to-palate distance at D1-D5			
PARAMETERS BEING CORRELATED	N	Correlation®	P VALUE
Degree of buccal tipping of 14 & Tongue to Palate distance at D1	63	0.404	0.001
Degree of buccal tipping of 14 & Tongue to Palate distance at D2	63	0.4	0.001
Degree of buccal tipping of 14 & Tongue to Palate distance at D3	63	0.502	<0.001
Degree of buccal tipping of 14 & Tongue to Palate distance at D4	63	0.492	<0.001
Degree of buccal tipping of 14 & Tongue to Palate distance at D5	63	0.359	0.004

**Table 3:** Correlations between the degree of buccal tipping at 27,26,25 and the tongue-to-palate distance at D1-D5.

Table 3 (A) Correlations between the degree of buccal tipping at 27 and the tongue-to-palate distance at D1-D5			
PARAMETERS BEING CORRELATED	N	Correlation®	P VALUE
Degree of buccal tipping of 27 & Tongue to Palate distance at D1	63	0.357	0.004
Degree of buccal tipping of 27 & Tongue to Palate distance at D2	63	0.357	0.004
Degree of buccal tipping of 27 & Tongue to Palate distance at D3	63	0.44	<0.001
Degree of buccal tipping of 27 & Tongue to Palate distance at D4	63	0.455	<0.001
Degree of buccal tipping of 27 & Tongue to Palate distance at D5	63	0.34	0.006
Table 3 (B) Correlations between the degree of buccal tipping at tooth 26 and the tongue-to-palate distance at D1-D5			
PARAMETERS BEING CORRELATED	N	Correlation®	P VALUE
Degree of buccal tipping of 26 & Tongue to Palate distance at D1	63	0.305	0.015
Degree of buccal tipping of 26 & Tongue to Palate distance at D2	63	0.268	0.034
Degree of buccal tipping of 26 & Tongue to Palate distance at D3	63	0.355	0.004
Degree of buccal tipping of 26 & Tongue to Palate distance at D4	63	0.354	0.004
Degree of buccal tipping of 26 & Tongue to Palate distance at D5	63	0.255	0.044
Table 3 (D) Correlations between the degree of buccal tipping at position 25 and the tongue-to-palate distance at D1-D5			
PARAMETERS BEING CORRELATED	N	Correlation®	P VALUE
Degree of buccal tipping of 25 & Tongue to Palate distance at D1	63	0.401	0.001
Degree of buccal tipping of 25 & Tongue to Palate distance at D2	63	0.409	0.001
Degree of buccal tipping of 25 & Tongue to Palate distance at D3	63	0.494	<0.001
Degree of buccal tipping of 25 & Tongue to Palate distance at D4	63	0.509	<0.001
Degree of buccal tipping of 25 & Tongue to Palate distance at D5	63	0.378	0.002
Table 3 (D) Correlations between the degree of buccal tipping at position 25 and the tongue-to-palate distance at D1-D5			
PARAMETERS BEING CORRELATED	N	Correlation®	P VALUE
Degree of buccal tipping of 24 & Tongue to Palate distance at D1	63	0.44	<0.001
Degree of buccal tipping of 24 & Tongue to Palate distance at D2	63	0.431	<0.001
Degree of buccal tipping of 24 & Tongue to Palate distance at D3	63	0.524	<0.001
Degree of buccal tipping of 24 & Tongue to Palate distance at D4	63	0.522	<0.001
Degree of buccal tipping of 24 & Tongue to Palate distance at D5	63	0.405	0.001

**Table 4:** Correlations between the degree of lingual tipping at position 37,36,35,34 and the tongue-to-palate distance at D1-D5.

Table 4 (A) Correlations between the degree of lingual tipping at position 37 and the tongue-to-palate distance at D1-D5			
PARAMETERS BEING CORRELATED	N	Correlation®	P VALUE
Degree of lingual tipping of 37 & Tongue to Palate distance at D1	63	0.337	0.007
Degree of lingual tipping of 37 & Tongue to Palate distance at D2	63	0.311	0.013
Degree of lingual tipping of 37 & Tongue to Palate distance at D3	63	0.361	0.004
Degree of lingual tipping of 37 & Tongue to Palate distance at D4	63	0.342	0.006
Degree of lingual tipping of 37 & Tongue to Palate distance at D5	63	0.259	0.041
Table 4 (B) Correlations between the degree of lingual tipping at position 36 and the tongue-to-palate distance at D1-D5			
PARAMETERS BEING CORRELATED	N	Correlation®	P VALUE
Degree of lingual tipping of 36 & Tongue to Palate distance at D1	63	0.327	0.009
Degree of lingual tipping of 36 & Tongue to Palate distance at D2	63	0.305	0.015
Degree of lingual tipping of 36 & Tongue to Palate distance at D3	63	0.345	0.006
Degree of lingual tipping of 36 & Tongue to Palate distance at D4	63	0.319	0.011
Degree of lingual tipping of 36 & Tongue to Palate distance at D5	63	0.26	0.04
Table 4 (C) Correlations between the degree of lingual tipping at position 35 and the tongue-to-palate distance at D1-D5			
PARAMETERS BEING CORRELATED	N	Correlation®	P VALUE
Degree of lingual tipping of 35 & Tongue to Palate distance at D1	63	0.323	0.01
Degree of lingual tipping of 35 & Tongue to Palate distance at D2	63	0.306	0.015
Degree of lingual tipping of 35 & Tongue to Palate distance at D3	63	0.349	0.005
Degree of lingual tipping of 35 & Tongue to Palate distance at D4	63	0.33	0.008
Degree of lingual tipping of 35 & Tongue to Palate distance at D5	63	0.276	0.028
Table 4 (D) Correlations between the degree of lingual tipping at 34 and the tongue-to-palate distance at D1-D5			
PARAMETERS BEING CORRELATED	N	Correlation®	P VALUE
Degree of lingual tipping of 34 & Tongue to Palate distance at D1	63	0.329	0.008
Degree of lingual tipping of 34 & Tongue to Palate distance at D2	63	0.305	0.015
Degree of lingual tipping of 34 & Tongue to Palate distance at D3	63	0.338	0.007
Degree of lingual tipping of 34 & Tongue to Palate distance at D4	63	0.322	0.01
Degree of lingual tipping of 34 & Tongue to Palate distance at D5	63	0.27	0.032

**Table 5:** Correlations between the lingual tipping degree of tooth 47,46,45,44 and the tongue-to-palate distance at D1-D5.

Table 5 (A) Correlations between the degree of lingual tipping of 47 and the tongue-to-palate distance at D1-D4			
PARAMETERS BEING CORRELATED	N	Correlation®	P VALUE
Degree of lingual tipping of 47 & Tongue to Palate distance at D1	63	0.324	0.01
Degree of lingual tipping of 47 & Tongue to Palate distance at D2	63	0.324	0.01
Degree of lingual tipping of 47 & Tongue to Palate distance at D3	63	0.331	0.008
Degree of lingual tipping of 47 & Tongue to Palate distance at D4	63	0.345	0.006
Table 5 (B) Correlations between the degree of lingual tipping in tooth 46 and the tongue-to-palate distance at D1-D5			
PARAMETERS BEING CORRELATED	N	Correlation®	P VALUE
Degree of lingual tipping of 46 & Tongue to Palate distance at D1	63	0.35	0.005
Degree of lingual tipping of 46 & Tongue to Palate distance at D2	63	0.311	0.013
Degree of lingual tipping of 46 & Tongue to Palate distance at D3	63	0.367	0.003
Degree of lingual tipping of 46 & Tongue to Palate distance at D4	63	0.311	0.013
Degree of lingual tipping of 46 & Tongue to Palate distance at D5	63	0.25	0.048
Table 5 (C) Correlations between the degree of lingual tipping at position 45 and the tongue-to-palate distance at D1-D4			
PARAMETERS BEING CORRELATED	N	Correlation®	P VALUE
Degree of lingual tipping of 45 & Tongue to Palate distance at D1	63	0.323	0.01
Degree of lingual tipping of 45 & Tongue to Palate distance at D2	63	0.293	0.02
Degree of lingual tipping of 45 & Tongue to Palate distance at D3	63	0.338	0.007
Degree of lingual tipping of 45 & Tongue to Palate distance at D4	63	0.31	0.013
Table 5 (D) Correlations between the lingual tipping degree of tooth 44 and the tongue-to-palate distance at D1-D5			
PARAMETERS BEING CORRELATED	N	Correlation®	P VALUE
Degree of lingual tipping of 44 & Tongue to Palate distance at D1	63	0.313	0.012
Degree of lingual tipping of 44 & Tongue to Palate distance at D2	63	0.285	0.023
Degree of lingual tipping of 44 & Tongue to Palate distance at D3	63	0.338	0.007
Degree of lingual tipping of 44 & Tongue to Palate distance at D4	63	0.312	0.013
Degree of lingual tipping of 44 & Tongue to Palate distance at D5	63	0.256	0.043

## Data Availability

The datasets used and/or analyzed during the current study are available from the corresponding author.
